# Subcellular localization of L-selectin ligand in the endometrium implies a novel function for pinopodes in endometrial receptivity

**DOI:** 10.1186/1477-7827-10-46

**Published:** 2012-06-15

**Authors:** Reza Nejatbakhsh, Maryam Kabir-Salmani, Eva Dimitriadis, Ahmad Hosseini, Robabeh Taheripanah, Yousef Sadeghi, Yoshihiro Akimoto, Mitsutoshi Iwashita

**Affiliations:** 1 Biology and Anatomy Department, Medical School, Shaheed Beheshti University of Medical Sciences, Tehran, Iran; 2 Molecular Genetics Department, National Institute of Genetic Engineering and Biotechnology, Tehran, Iran; 3 Cellular and Molecular Biology Research Center, Medical School of Shaheed Beheshti University of Medical Sciences, Tehran, Iran; 4 Embryo Implantation Laboratory, Prince Henry’s Institute of Medical Research, Melbourne, Australia; 5 Infertility and Reproductive Health Research Center, Shaheed Beheshti University of Medical Sciences, Tehran, Iran; 6 Department of Anatomy, Kyorin University School of Medicine, Tokyo, Japan; 7 Department of Obstetrics and Gynecology, Kyorin University School of Medicine, Tokyo, Japan

**Keywords:** Endometrium, Uterodome, Implantation, MECA-79, L-selectin Ligand, Pinopode

## Abstract

**Background:**

Apical surfaces of human endometrial epithelium and endothelium are key elements for the initiation of molecular interactions to capture the blastocyst or leukocyte, respectively. The L-selectin adhesion system has been strongly proposed to play an important role in the initial steps of trophoblast adhesion and promotion of integrin-dependent processes, ultimately culminating in the establishment of the embryo-maternal interface. On the basis of these facts, we hypothesized a novel role for pinopodes as the first embryo-fetal contact sites to contain the highest subcellular expression of L-selectin ligand suggesting its role in early adhesion as predicted. Thus, the objective of this study was therefore to determine the subcellular pattern of distribution of the L-selectin ligand (MECA-79) in human endometrial apical membrane region during the window of implantation.

**Methods:**

Endometrial biopsies of secretory phases from fertile females ranging in age between 25 and 42years were studied using several approaches, including scanning electron microscopy (SEM), immunostaining for light microscopy and transmission electron microscopy (TEM), and immunoblotting as well as statistical analysis of the area-related numerical densities of immunoreactive MECA-79-bound nanogolds to detect the expression pattern and the subcellular distribution pattern of L-selectin ligand (MECA-79) in human endometrium during the window of implantation.

**Results:**

The endometrial biopsies were scored according the dating criteria of Noyes et al. by an experienced histologist. The SEM images of the midluteal phase specimens revealed that fully developed pinopodes were abundant in our samples. HRP-immunostaining and immunofluorescent staining as well as immunoblotting revealed that MECA-79 was expressed in the midluteal phase specimens. The results of immunogold TEM illustrated the expression of MECA-79 in human pinopodes in the midluteal phase and a higher area-relate numerical density in pinopodes compared to that of the uterodome-free areas.

**Conclusions:**

This is the first demonstration of the subcellular localization of MECA-79 in the human pinopodes which may indicate a novel role for pinopodes to be capable of shear-stress-dependent tethering-type adhesion in the initial phases of human embryo implantation.

## Background

In humans, there is a distinct ‘window of implantation’ during the midluteal phase when the endometrium demonstrates maximal receptivity for embryo implantation [1]. There is convincing data that the uterine luminal epithelium is central in controlling receptivity, and probably acting initially as a barrier to challenge attaching embryo [2]. Several candidate adhesion molecules are expressed in the luminal epithelium of receptive endometrium where they are thought to facilitate embryo apposition/adhesion/ communication to the endometrium [3-5]. However, because of difficulties in analyzing these processes in human models, differences among primate species [6,7], the lack of suitable experimental models, and significant variations between rodent and human models of embryo implantation [1], the exact molecular basis of these initial interactions remain to be discovered.

Considering the fact that the human embryo must attach itself to the uterus under conditions of shear stress similar to that of leukocyte transmigration, numerous investigators have proposed that the molecular basis between implantation and leukocyte transmigration should bear some similarities. Several investigators have demonstrated that L-selectin and its oligosaccharide ligands, which constitute L-selectin adhesion system, could be considered as one of the most important candidate pathways to mediate the initial embryo-maternal interactions [8,9]. In support, it was recently demonstrated that human blastocysts express L-selectin on their external surfaces [10] raising the possibility that these molecules may participate in the early stages of human blastocyst attachment. Furthermore, the lack of detection of L-selectin at stages earlier than the embryonic blastocyst stage and L-selectin’s intense immunostaining on the trophectoderm during hatching, indicates that this cell surface protein is developmentally regulated [11]. This was correlated with complementary increased expression of its ligand within the luminal epithelium during the mid-secretory phase [10,12]. Immunolocalization studies on normal endometrium have demonstrated that the L-selectin carbohydrate ligand MECA-79, antibody that recognizes L-selectin high-affinity ligands, is up-regulated from the day of ovulation to day 6-post ovulation, and by comparison is reduced throughout the follicular phase or in ovulatory cycles [10,12,13]. More interestingly, a significant difference between the expression of L-selectin ligands between fertile and infertile women was reported and the MECA-79 epitopes were shown to be expressed at a higher level in fertile compared to that of infertile patients [14,15]. Other than embryo implantation [10,16], this adhesion system correlates with many physiological and pathological processes including leukocyte infiltration [17,18], lymphocyte homing [19], and tumor metastasis [20,21] indicating the existence of similar pathways between these processes. Since the apical surface of the endometrium contains key elements for the initiation of molecular interactions between human blastocysts and the endometrium, and that the L-selectin ligand adhesion system is believed to play a major role in mediating initial embryonic apposition and adhesion, this prompted us to investigate the subcellular localization of L-selectin ligand at the pinopodes, the most apical surfaces of the receptive endometrium [22], and the first embryo-maternal contact sites. In the present study, we proposed that pinopodes may have a novel function in the unusual cell-cell interactions that take place in the early steps of human embryo-endometrial apposition/adhesion similar to that of tethering of blood cells.

## Methods

### Endometrial biopsy specimens

Endometrial biopsies were obtained during secretory phases were collected by curettage from the anterior wall of the uterine cavity of women (ages 25–42) undergoing minor gynecological investigations unrelated to endometrial pathology. All women were fertile with regular menstrual periods (25–35days) and none of them had used steroidal contraceptives or intrauterine devices for at least 3months prior to the sampling. All patients gave their informed consent for the collection and investigational use of endometrial tissues. This study was approved by the local Ethical Committee and Southern health Human Research Ethics Committee, (#09317B), Monash Medical Centre, Clayton, Victoria (cell culture experiments) and Shaheed Beheshti Medical Sciences University Ethical Committee, (#88-01-115-6367-3144), Tehran-Iran (all other experiments). Eight patients were tested for our experiments, 3 patients for immunoblotting and 5 patients for all other experiments. Except immunoblotting biopsies, another biopsies from each patient were divided to several small portions to be used for light microscopy, immunohistochemistry, scanning electron microscopy (SEM) and immunogold staining of transmission electron microscopy (TEM) experiments. For endometrial dating, samples were fixed in 10% neutrally buffered formaldehyde and the paraffin-embedded biopsies were stained with hematoxylin and eosin and evaluated by an experienced observer according to the histopathological criteria of Noyes [23]. Samples used for cell culture and western blot analysis were collected in a 1:1 mixture of DMEM and Ham’sF12 medium (DMEM/F12; Trace Biosciences, Sydney, Australia) supplemented with 1% penicillin, streptomycin, and fungizone (Common Wealth Serum Laboratories, Melbourne, Australia) and L-glutamine (Sigma Diagnostics, St. Louis, MO).

### Culture of primary human endometrial epithelial cells and human endometrial epithelial (HES) cell line

Fresh samples were transferred in a 1:1 mixture of DMEM/F12 supplemented with 1% penicillin, streptomycin, and fungizone and L-glutamine to culture room and washed three times. Human endometrial tissues were digested with collagenase type 3, 0.2mg/ml for 20min 2 times and the suspension was filtered through 43 and 11μm nylon mesh to collect endometrial epithelial fragments, as previously described work [24]. Briefly, the epithelial fragments were collected and re-suspended in a 1:1 mixture of DMEM/Hams F-12 (F12; Thermo Electron Corp., Melbourne, Australia) supplemented with 10% fetal calf serum (FCS; Invitrogen, Carlsbad, CA), 2mML-glutamine (Thermo Electron), and 1% antibiotic antimycotic solution (Life Technologies, Inc., Auckland, New Zealand) and plated. Endometrial epithelial cells were collected from the filter paper and cells were further purified by selective adherence. Epithelial cells were allowed to grow out from glandular structures for 48h, then detached with trypsin, and serially replated (three times) in plastic culture dishes for 30min each time, to allow adherence of contaminating stromal cells. Non-adherent cells were transferred to 24-well plates and were allowed to grow out for 48h. Cells were grown in DMEM/F12/charcoal-stripped fetal calf serum for 48h until 80% confluent. This method achieves cultures of 85–90% epithelial cell purity, as judged by morphological and immunohistochemical criteria [25]. A pan-cytokeratin antibody (Dako) diluted 1:4 in non-immune block after enzymatic antigen retrieval (0.1% trypsin in 0.1% CaCl2 for 15min at 37°C) were used for immunohistochemical staining.

For the extended experiments in which insufficient primary EECs were available, a human endometrial epithelial (HES) cell line were used. The HES cell line [26] was obtained from Dr. Douglas Kniss (Ohio State University, Columbus, OH). Cells were maintained in RPMI 1640 (Thermo Electron) supplemented with 10% FCS. Confluent cells were transferred into serum reduced (1% FCS) medium for 24h before use.

### Scanning electron microscopy (SEM)

SEM was performed to evaluate the presence of pinopodes in the endometrial specimens obtained at the midluteal phase of the menstrual cycle. For SEM preparation, endometrial biopsies were fixed for at least 24h in 2.5% glutaraldehyde in 0.1M phosphate buffer (pH 7.4) at 4°C and postfixed using 1% OsO_4_ in 0.1M phosphate buffer (pH 7.4) for 1h. The specimens were then dehydrated in a graded series of ethanol (50%, 70%, 90%, 99.5% and 100%), critical-point-dried with carbon dioxide using a freeze drying device (JFD–300, JEOL, Tokyo, Japan), mounted, and coated with gold in a sputter coater. Finally, the specimens were observed under a scanning electron microscope (JSM- 5600 LV SEM, JEOL).

### Immunohistochemistry and immunofluorescent staining for light microscopy

Polymer peroxidase staining (Envision+/HRP; DakoCytomation, Denmark) was performed as described previously [27]. Briefly, using optimal cutting temperature (OCT) compound-embedded samples, cryostatic sections (4μm thickness) were mounted on positively charged slides, fixed with ice-cold 100% acetone for 10 minutes and air-dried. After blocking endogenous peroxidase activity by immersing the slides in 0.3% hydrogen peroxide (H_2_O_2_) in methanol for 10 minutes at room temperature, nonspecific background was blocked with 5% BSA for 30 minutes at room temperature. The tissue sections were then incubated overnight at 4°C with an anti-human L-selectin ligand monoclonal antibody (MECA-79; BD Pharmingen) at a concentration of 2μg/ml and isotype IgG control at the same concentration as the primary antibody. After washing, sections were incubated with an Envision+/HRP anti-mouse secondary IgG for 30min at room temperature. Peroxidase activity was detected by incubating the specimens for about 10min at room temperature with 0.5mg/ml DAB-0.005% H_2_O_2_-PBS. Finally, nuclear counterstaining was done with hematoxylin. A positive control section of tonsil and a negative (no antibody) control section of endometrium tissue were used to determine the specificity of the antibody.

For immunofluorescent staining, cryostatic sections were fixed with ice-cold 100% acetone for 10 minutes and air-dried. Nonspecific background was then blocked with 5% BSA for 30 minutes at room temperature. The tissue sections then were incubated overnight at 4°C with 2μg/ml of an anti-human L-selectin ligand monoclonal antibody (MECA-79). For negative controls, normal mouse serum IgG was used instead of primary antibody. The tissue sections were then counter-stained with appropriate secondary antibody (FITC-labeled IgG, 2μg/ml) and incubated for 1–3h at room temperature. Tissue sections were washed with PBS, rinsed in deionized water and mounted. The tissue sections were then observed using an AX-80 fluorescence microscope (Olympus Optical).

### Immunostaining for transmission electron microscopy (TEM)

Immunogold staining for TEM was performed to determine the ultrastructural distribution of L-selectin ligand (MECA-79) according to previous reports [27]. Briefly, specimens were divided into the 2mm^3^ blocks and fixed in 4% PFA in 0.1M phosphate buffer (pH 7.4) for at least 24h at 4°C. After dehydration in a graded series of ethanol (50%, 70%, 90%, 99.5%, 100%), they were embedded in Lowicryle white resin (London Resin company Ltd., London, UK). They were cut into ultrathin sections, which were then washed in PBS and pretreated with 5% BSA for 10min at room temperature. After rinsing in PBS, they were incubated overnight at 4°C with a monoclonal anti-human L-selectin ligand antibody (MECA-79, 1.5μg/ml) or with normal mouse sera (1.5μg/ml), as negative control. Following washing in PBS (5 times, 5min each), the ultrathin sections were incubated overnight at 4°C with 12nm colloidal gold-conjugated secondary (Jackson Immuno Research Laboratories Inc., West Grove, PA, USA), diluted with PBS (1:20). The ultrathin sections were then washed in PBS followed by washing in distilled water. The ultrathin sections were stained with uranyle acetate and observed under a transmission electron microscope (JEM-1010; JEOL, Japan).

Morphometric and statistical analyses were performed to determine the expression pattern of MECA-79 during the opening of the implantation window, that is, days 19–20 of a normal menstrual cycle. For morphometric analysis, 400 fields obtained at the midluteal phase (40 fields for each biopsy specimen, each field equaled to 8.04μm^2^) were randomly chosen near the cell membrane of either pinopodes or the neighboring uterodome-free areas by an observer who was blind to the identity of the grids. Then, the number of immunogold particles was counted in all selected areas at the same magnification (X15000). After calculating the area-related numerical densities of immunogold particles, statistical analysis was performed comparing pinopodes and the neighboring uterodome-free areas. For the morphometric assessment of the area-related numerical densities of immunogold-conjugated MECA-79, statistical analysis was carried out by taking the mean number of immunogold particles in 40 fields per block of each specimen from 5 patients’ endometrial biopsies. The area-related numerical density of immunogold particles was expressed as Mean±SEM. Statistical significance was evaluated using paired sample t- test and a *p*<*0*.*05* was considered statistically significant.

### Immunoblotting

Immunoblotting was performed for expression studies of MECA-79. Both endometrial epithelial cell line (HES) and primary human endometrial epithelial cells (hEEC) were used and western blot analyses were performed using standard protocols as previously described [28]. Briefly, equal amounts of protein lysate (10μg) were separated on 8.5% SDS/PAA gels and transferred onto polyvinylidene difluoride membranes (Hybond-P; Amersham Pharmacia Biotech, Piscataway, NJ). All membranes were incubated with Ponceau-S (Sigma) to ensure equal protein loading in all lanes. After blocking with 5% nonfat dry milk in TBS with 0.1% Tween-20 (Bio-Rad Laboratories, Hercules, CA), membranes were incubated overnight (4°C) with rat monoclonal antibody MECA-79 (200μg/0.1ml, 1:500, Santa Cruz, SC 19602L). After washing with 0.2% Tween-20/TBS, the membranes were incubated 1h (room temperature) with secondary antibodies (rabbit anti rat IgG horseradish peroxidase linked, Cell Signaling Technology, Beverly, MA, 1:2500) signals were developed by using ECL Western blotting detection system (Pierce, Rockford, IL, USA) followed by the exposure of the membranes to a Kodak X-AR film (Eastman Kodak co., Rochester, NY, USA) for 1–5min at room temperature. To analyze nonspecific secondary antibody binding, membranes were stripped and incubated with isotype control rat IgG (Sigma, Chemical Company, St. Louis, MO, 1.7mg/ml). Page Ruler prestained protein ladder (Fermentas, St. Leon-Rot, Germany) was used as a molecular size marker. This experiment was performed in triplicate using three different samples obtained at the midluteal phase.

## Results

**Figure 1:**
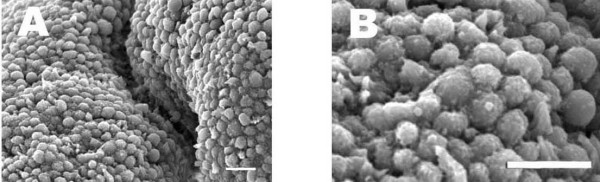
**SEM photomicrograph from the human endometrium in the mid- secretory phase of the menstrual cycle. A** and **B** showed Low and high magnification of human endometrium in the mid- secretory phase of the menstrual cycle, respectively. Note the numerous fully developed pinopodes. Scale bar=10μm. Representative photo of 5 tissues.

SEM images demonstrated that the endometrial luminal epithelium in the midluteal phase of the menstrual cycle showed two different types of cells: ciliated and nonciliated cells. The majority of the luminal epithelial cells were of the latter type (Figure1 A and B). The membranous projections on the apical pole of nonciliated cells appeared as fine microvilli and were dome-like. The SEM images of the midluteal phase specimens revealed that fully developed pinopodes were abundant in these samples among few regressing pinopodes (Figure1 A and B).

**Figure 2:**
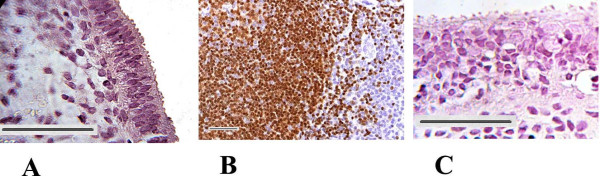
**Immunolocalization of L-selectin ligand.** Positive staining for L-Selectin ligand showed as brown pigment. **(A)** Mid-secretory phase of human endometrium, **(B)** tonsil as positive control **(C)** negative control. Scale bars=50μm. Representative photo of 5 tissues.

Strong immunoreactivity for MECA-79 was seen in the midluteal phase human endometrium (Figure2A). MECA-79 localized at the luminal and glandular epithelium predominantly at the cell membrane with little cytoplasmic staining observed. By contrast, no MECA-79 immunostaing was visible in the stroma. Section of tonsil was used as positive control demonstrated immunoreactivity for MECA-79 (Figure2B). In the negative controls, no staining was observed (Figure2C).

**Figure 3:**
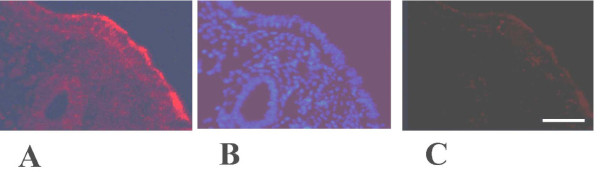
**Immunofluorescent images of L-Selectin ligand.** Human endometrial biopsies obtained from midluteal phase of a normal menstrual cycle. Red colour showed L-Selectin ligand positive fluorescent **(A)** Blue colour demonstrated nucleus staining by DAPI **(B)**, negative control **(C)**. Scale bar=50μm and representative for Figures A-C. A-C is representative photos of 5 tissues.

Similarly immunofluorescent staining revealed MECA-79 localized predominantly at luminal epithelium in the midluteal phase human endometrium (Figure3A). In the negative controls, no staining was observed (Figure3C).

**Figure 4:**
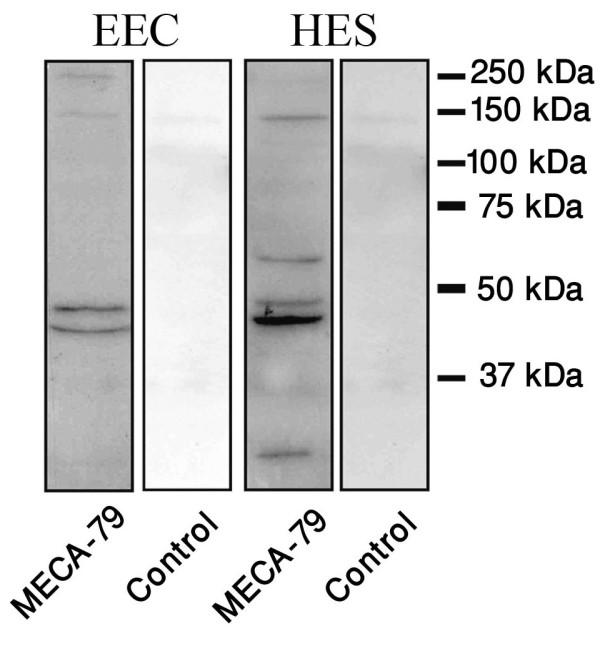
**Immunoblot analysis for L-selectin ligand.** Endometrial epithelial cell line (HES) and primary human endometrial epithelial cells (EEC) is used for this experiment. In the control groups, rat IgG was used instead of primary antibody to analyze nonspecific secondary antibody binding. Data is shown as a single photomicrograph representative of 3 independent experiments.

Immunoblotting analysis demonstrated the expression of L-selectin ligand in both epithelial endometrial cells (EEC) and human endometrial epithelial cell line (HES) in protein level (Figure4), while no expression was seen in negative controls using rat IgG as isotype control.

**Figure 5:**
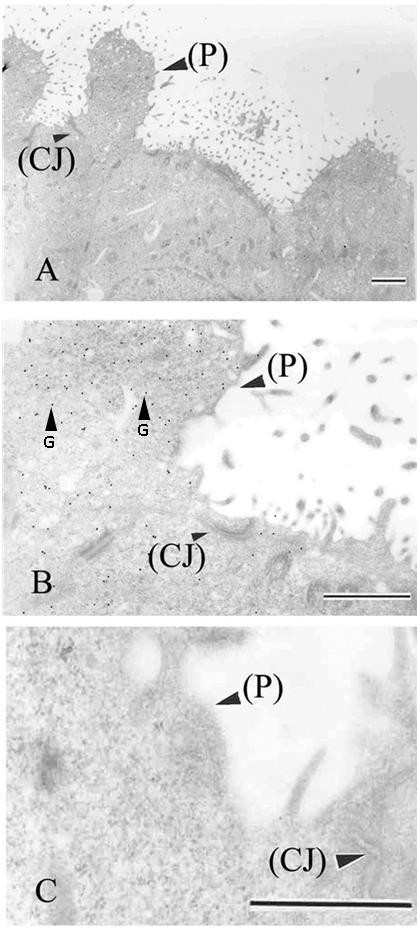
**Images of immunogold transmission electron microscopy (TEM) labeling for L-selectin ligand.** Human endometrial biopsies obtained from midluteal phase of a normal menstrual cycle. Low **(A)**, high **(B)** magnification and negative control **(C)**. P, pinopode; CJ, connective junction: G, Gold. Scale bars=1μm. Representative photo of 5 tissues.

In the immunogold TEM photomicrographs, MECA-79-conjugated nanogolds were predominantly observed in the pinopodes (Figure5A, 5B), implicating a different subcellular area-related expression pattern of MECA-79 in the luminal epithelial cells of the midluteal phase endometrium (Figure5B). In the negative controls, no staining was observed (Figure5C).

**Figure 6:**
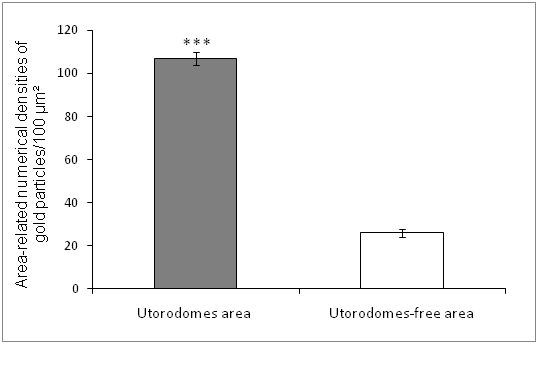
**Statistical analysis of the area- related numerical densities of immunogold conjugated L-Selectin ligand in human mid-secretory phase endometrial biopsies.** The statistical analyses of the number of immunogold particles revealed that the distribution of L-Selectin ligand in these specimens was significantly (p<0.001) higher in the pinopodes of the mid-secretory phase specimen compared to pinopode- free areas. ***: p<0.001.

Semi-quantitative statistical analysis revealed that the area-related numerical densities of the MECA-79-conjugated nanogolds were 3.9 higher in pinopodes compared to that of pinopode-free neighboring areas (Figure6).

## Discussion

To the best of our knowledge, this is the first report of the subcellular localization of L-selectin ligand MECA-79 in the human pinopodes suggest a novel role for pinopodes possibly in inducing a shear-stress-dependent tethering-type adhesion in the initial phases of human embryo implantation. It has been reported that pinopodes are integrin-enriched subcellular structures that can be considered as biomarkers of endometrial receptivity [29]. However, the clinical usefulness of pinopodes to delineate a period of endometrial receptivity seems unlikely following recent findings that pinopodes have a prolonged (>5days) presence in the luteal phase and fail to delineate the brief (24–48h) window of receptivity [30]. Therefore, the functional importance of pinopodes, bleb-like hormone-dependent structures that appear at the time of implantation and extend beyond the glycocalyx layer of the apical membrane of the endometrial epithelium, remains elusive [31,32]. Endometrial pinopodes resemble morphologically the docking structures of the endothelium [22] that are believed to be key elements for the initiation of molecular interactions to capture the blastocyst or leukocyte, respectively, indicating an active role for adhesive three dimensional docking structures in the extravasation sequence corresponds to the interaction of selectins with their carbohydrate-based ligands [33-35]. Thus, an objective of this study was to detect the existence of a member of such systems at pinopodes.

To this end, MECA-79, a monoclonal antibody that blocks L-selectin-dependent lymphocyte attachment and recognizes 6-sulfo sLex, a sulfation dependent determinant on L-selectin ligands [36,37] was used to determine the subcellular distribution pattern of the L-selectin ligand in human endometrial epithelial cells and in pinopodes. The antibody binds the sulfated oligosaccharide epitopes of L-selectin ligands, enabling the quantification of L-selectin expression in represented tissue samples [38,39]. The evaluation of the expression pattern of L-selectin ligand was based on a semi quantitative immunohistochemical score, incorporating morphometric analysis of area-related numerical density of MECA-79-conjugated nanogolds in pinopode and adjacent pinopode-free areas of apical cell membrane of luminal endometrial epithelium. Meca-79 immunostainings demonstrated that MECA-79 localized at the luminal and glandular epithelium predominantly at the cell membrane with little cytoplasmic staining observed (Figure2, panel b). By contrast, no MECA-79 immunostaing was visible in the stroma. This is inconsistent with a previous report which showed that in the glandular epithelium the expression of L-selectin ligands was greatest in the midluteal phase [12]. Western blot analysis also showed that L-selectin ligand protein was detected in endometrial epithelial culture of our endometrial epithelial cell line (HES) as well as in primary endometrial epithelial cells. These results demonstrated the presence of L-selectin ligand in the human endometrial epithelial cells, supporting our immunostaining results and were consistent with pervious publications in cultured endometrial epithelial cells [10,15]. Moreover, the results of our TEM immunogold staining demonstrated the expression of MECA-79 at pinopodes of mid-secretory endometrial biopsies. The semi quantitative morphometric analysis revealed that statistically higher area-related numerical density of MECA-79-conjugated nanogolds exists in pinopode compared to that of adjacent pinopode-free areas. This is, to our knowledge, the first report of subcellular and distribution pattern of L-Selectin ligand in human luminal endometrial epithelium. These results can propose a novel role for human endometrial pinopodes similar to that of endothelial docking structures in tethering process which allows the leukocyte to roll on the endothelial cell wall. It seems that the L-selectin adhesion system plays a role in human, but not mouse embryo implantation. It is reasonable that mutant mice deficient in the L-selectin gene show no defect in implantation even though a microarray analysis of mouse blastocysts has showed an elevation of L-selectin transcripts during the maturation stage, when the blastocysts are competent for implantation [40]. Thus, it might be concluded that other selectins or integrin ligands compensate for this deficiency in mice or the role of L-selectin in implantation is restricted to humans.

E-, L-, and P-selectin were originally thought to be expressed exclusively by hemangioblast descendents [9] and the discovery that the L-selectin system might function during reproduction was an unexpected finding. Our data together with previous reports may reveal that a clear parallelism between the different steps in human embryo-endometrial apposition/adhesion and leukocyte-endothelium rolling/adhesion can be established both at the molecular and morphological levels. Such homotypic adhesive interaction using the L-selectin adhesion system has been previously suggested by the other groups for placental cytotrophoblast [41,42]. They have a remarkable ability to modulate their adhesion molecule repertoire as they move through cell columns and acquire the ability to invade the uterine wall and the blood vessels that traverse this region. Of course, a large force is required to physically immobilize a free-floating cell to a flat surface. Given the enormous difference in size between a human blastocyst (diameter, 115~265mm) and lymphocyte (diameter, 10mm), it is difficult to imagine that a blastocyst could be immobilized to endometrial epithelia solely through L-selectin, given the somewhat weak selectin-carbohydrate interactions [43,44]. It seems reasonable to speculate that a human blastocyst rolls over the glycocalyx of the endometrial epithelium through weak interactions with L-selectin, similar to the movement of lymphocytes over endothelial cells that is mediated by L-selectin. L-selectin-mediated rolling may allow cross-talk between the blastocyst and maternal epithelia, leading to stronger cell adhesion by direct binding between the components embedded in the plasma membranes on the fetal and maternal sides. Considering the report that showed pinopode formation is accompanied by loosening of endometrial inter epithelial cell contacts strengthens our hypothesis that the expression of L-selectin ligand in pinopodes and the activation of L-selectin ligand-mediated rolling might facilitate stronger blastocyst attachment and penetration to the lateral membrane of endometrial epithelial cells. This is consistent with a previous suggested model for human embryo adhesion phases, termed as early and late adhesion events [45]. Moreover, previous studies showed that mucin-1 (Muc-1), a cell-surface glycoprotein expressed on pinopodes, carries both sLex and MECA-79 epitopes [46,47], although another study indicates that MUC1 is expressed on ciliated cells [48]. As the presence of L- selectin ligand is not restricted to the ciliated cells [10,12,13,15], it seems that MUC1 is one of the carriers of L- selectin ligand in human endometrium [45]. MUC1 is a large, transmembrane mucin glycoprotein abundantly expressed at the apical surface of uterine epithelia in all species examined to date. Loss of MUC1 at the time of embryo implantation occurs in many species; however, this does not appear to be the case in humans, suggesting that the human endometrium actively prevents the embryo from adhering except at the very spot of implantation. Selectins are proposed to have an important role in this phase to ensure suitable rolling of the blastocyst to ensure that the blastocyst will settle in the proper position and in the correct orientation.

## Conclusions

In conclusion, this study highlights the potential importance of pinopodes in the early steps of human embryo-endometrial apposition/adhesion and identities a possible novel role for pinopodes in human implantation. It provide evidence for the concept that trophoblast/ endometrium may use the L-selectin adhesion system to carry out a type of tethering adhesion that facilitates homotypic rather than heterotypic interactions demonstrating a relative abundance of L-selectin ligands presented in endometrial pinopodes.

## Competing interests

The authors declare that they have no competing interests.

## Authors’ contributions

RN performed cell cultures, Western blot, immunohistochemistry, and assisted in drafting the manuscript. MKS and ED conceived the study, designed, and participated in data analysis and drafted the manuscript. AH and MI designed and provided assistance in co-ordination of the study. RT prepared ethical approval, recruited patients and collected samples**.** YA and YS developed the SEM and TEM assays**.** All authors critically analysed the manuscript and approved the final manuscript.
