# Correction to: Subcellular localization of L-selectin ligand in the endometrium implies a novel function for pinopodes in endometrial receptivity

**DOI:** 10.1186/s12958-021-00745-w

**Published:** 2021-04-23

**Authors:** Reza Nejatbakhsh, Maryam Kabir-Salmani, Evdokia Dimitriadis, Ahmad Hosseini, Robabeh Taheripanah, Yousef Sadeghi, Yoshihiro Akimoto, Mitsutoshi Iwashita

**Affiliations:** 1 Biology and Anatomy Department, Medical School, Shaheed Beheshti University of Medical Sciences, Tehran, Iran; 2 Molecular Genetics Department, National Institute of Genetic Engineering and Biotechnology, Tehran, Iran; 3 Cellular and Molecular Biology Research Center, Medical School of Shaheed Beheshti University of Medical Sciences, Tehran, Iran; 4 Embryo Implantation. Laboratory, Prince Henry’s Institute of Medical Research, Melbourne, Australia; 5 Infertility and Reproductive Health Research Center, Shaheed Beheshti University of Medical Sciences, Tehran, Iran; 6 Department of Anatomy, Kyorin University School of Medicine, Tokyo, Japan; 7 Department of Obstetrics and Gynecology, Kyorin University School of Medicine, Tokyo, Japan

**Correction to: Reprod Biol Endocrinol 10, 46 (2012)**

**https://doi.org/10.1186/1477-7827-10-46**

Following publication of the original article [1], the authors reported an error in the third author’s given name. The correct name should be Evdokia Dimitriadis.
